# FAM84B promotes the proliferation of glioma cells through the cell cycle pathways

**DOI:** 10.1186/s12957-022-02831-8

**Published:** 2022-11-23

**Authors:** Deshuai Ren, Xiaoyu Zhuang, Yanxin Lv, Yun Zhang, Jiazhi Xu, Fengquan Gao, Dagang Chen, Yu Wang

**Affiliations:** 1 Department of Neurosurgery, the Third Affiliated Hospital of Qiqihar Medical University, Qiqihar, 161000 China; 2 Hospital chief office, Tailai people’s Hospital, Qiqihar, China; 3 Department of Basic Medicine, Qiqihar Medical University, No. 333 Bukuibei Street, Jianhua District, Qiqihar, 161006 Heilongjiang Province China

**Keywords:** Glioma, FAM84B, Proliferation, Cell cycle

## Abstract

**Background:**

This study aimed to investigate FAM84B expression in glioma tissues and explore the role of FAM84B in promoting the proliferation of glioma cells and the mechanism of regulating the cell cycle pathways.

**Methods:**

The TCGA database was adopted to analyze FAM84B expression in glioma tissues. The FAM84B expression was detected by qRT-PCR in patients with glioma, especially that in glioma cells, U251, LN-229, U98, and U87. Two glioma cell lines U87 and T98 were selected for siRNA transfection, which were divided into si-NC si-FAM84B-1 and si-FAM84B-2 groups. The effect of FAM84B on the proliferation of glioma cells was detected with the MTT experiment and that on the glioma cell cycle was detected with the flow cytometry. The signaling pathways potentially regulated by FAM84B in glioma were analyzed through the bioinformatics analysis. The expression of proteins, Cyclin D1, CDK4, Cdk6, and p21, in the cell cycle-related pathways in cells of each group was detected by the Western blot.

**Results:**

TCGA database results showed a significantly higher FAM84B expression in glioma tissues than that in paracancerous tissues. According to the detection of qRT-PCR, FAM84B expressed the highest in the glioma cell line U87 (*P* < 0.05). Compared with the serum of healthy controls, FAM84B mRNA expression significantly increased in patients with gliomas. And compared with the si-NC group, the proliferation ability of U87 and T98 cells decreased and the cell cycle was blocked in the G0/G1 phase in both si-FAM84B transfection groups (*P* < 0.05). According to the bioinformatics analysis, FAM84B regulated the cell cycle pathways in glioma. FAM84B siRNA inhibited the expression of key proteins, Cyclin D1, CDK2, CDK4, and Cdk6, of the cell cycle pathways in glioma cells and promoted the expression of P53 and P21 proteins.

**Conclusions:**

In conclusion, FAM84B may inhibit the proliferation of glioma cells by regulating the cell cycle pathways.

## Research highlights

1. FAM84B expressed highly in glioma tissues and cells.

2. Knockdown of FAM84B expression significantly inhibited the proliferation of glioma cells.

3. Knockdown of FAM84B inhibited the proliferation of glioma cells by regulating the cell cycle signaling pathways.

## Introduction

Glioma is the most common primary intracranial tumor, which is considered as the most common malignancy affecting the central nervous system in adults [1]. Current standard therapies for glioma include surgical resection, radiotherapy, chemotherapy, and combination therapy [2]. However, glioma has the characteristics of rapid proliferation and strong diffusion and infiltration, which limits the feasibility of surgical resection. High drug resistance and recurrence rate lead to poor prognosis in patients with gliomas [3, 4], with the median overall survival of only 15 months [5]. According to the Chinese Glioma Genome Atlas, the median survival of Chinese patients with glioblastoma is only 14.4 months [6]. At present, the pathogenesis of glioma has not been fully defined, so it is of great significance to explore the molecules that alter and play a key role in the malignant progression of glioma to find effective treatment strategies for glioma.

Family with sequence similarity 84, member B, (FAM84B) with sequence similarity is one of the newly identified oncogenes closely related to tumorigenesis and development. The studies by Wong et al. [7] have reported that FAM84B significantly enhances the xenograft tumor and lung metastasis of prostatic cancer cells, to promote the malignant progression of prostatic cancer. Long non-coding RNA FAM84B-AS enhances the drug resistance to platinum in gastric cancer by inhibiting FAM84B expression, so as to promote the growth of gastric cancer cells [8]. Zhang et al. [9] reported that FAM84B promotes tumor progression in pancreatic ductal adenocarcinoma through the Wnt/β-catenin signaling pathway. Wang et al. [10] also reported that FAM84B expression is elevated in glioma specimens, which is associated with low survival in patients, and the silencing of FAM84B can inhibit the proliferation and invasion of glioma cells by regulating the Akt/GSK-3β/β-catenin pathway, thus inducing the apoptosis of glioma cells. This study adopted the bioinformatics analysis to find whether FAM84B could regulate the cell cycle signaling pathways in glioma and whether FAM84B plays an important role in the malignant progression of glioma through regulating the cell cycle signaling pathways in glioma. This study also tried to provide an important laboratory basis for FAM84B as a potential molecular target for the treatment of glioma.

## Experimental methods

### Bioinformatics analysis of FAM84B expression and mechanism

TCGA GBM RNA-Seq data were downloaded from https://xenabrowser.net using the UCSC Xena Browser. The differences in gene expression of FAM84B in glioma and control groups were analyzed, and the ROC curves of FAM84B gene expression were plotted using the R package “pROC.” In addition, the CPTAC module in the UALCAN database (http://ualcan.path.uab.edu/) was used to view the difference in protein levels of FAM84B between glioma and control groups. The level of immune invasion in gliomas was calculated using the ssGSEA method and the correlation of FAM84B gene expression with 28 immune cells was analyzed.

Select the Gene Analysis Module in the TISCH database (http://tisch.comp-genomics.org), enter FAM84B, and select Glioma to view FAM84B expression in various cell types. In addition, select the “Glioma_GSE89567” dataset in the data module to view the cell distribution, and enter the gene set of KEGG’s cell cycle pathway to view the difference in cell cycle pathway fraction in different cells.

The TCGA GBM RNA-Seq data was used to perform a Bayesian *t*-test by dividing the FAM84B median expression into high and low expression groups by the R package “limma.” KEGG pathway enrichment analysis was performed based on the R package “GSEA,” and values with *P* less than 0.05 were considered statistically significant. The cell cycle pathway score in glioma was calculated using the ssGSEA method, and the correlation between FAM84B gene expression and cell cycle pathway score was analyzed.

### Main reagent materials

Glioma cells, U251, T98, LN-229, and U87, were purchased from the Cell Bank of the Chinese Academy of Sciences; cell culture medium, trypsin, and FBS were purchased from Zhejiang Tianhang Biotechnology Co., Ltd.; penicillin/streptomycin was purchased from Sigma, USA; TRIzol reagent was purchased from Invitrogen Reagent, USA; AMV one-step RT-PCR kit and FAM84B and β-Actin primers were purchased from Sangon Biotech (Shanghai) Co., Ltd.; FAM84B siRNA was purchased from Guangzhou Ruibo Reagent Company; MTT reagent, cell cycle detection kit, and dual-luciferase assay kit were purchased from Shanghai Beyotime Biotechnology Inc.; ELISA apoptosis detection kits were purchased from Roche Reagent, Switzerland; protein lysate was purchased from Beijing Solarbao Science & Technology Co., Ltd.; BCA protein concentration detection reagent was purchased from Shanghai Biotrive Sci&Tech. Co., Ltd.; ECL chemiluminescence kits were purchased from Thermo, USA; Wnt3a (ab219412), β-catenin (ab68183), cMYC (ab32072), Cyclin D1 (ab16663), and GAPDH (ab9485) antibodies were purchased from Abcam, UK.

### Clinical samples

Blood samples from 40 patients with glioma admitted to our hospital from January 2013 to December 2019 were selected according to the inclusion criteria, as follows: patients who did not undergo radiotherapies, and chemotherapies; patients with glioma confirmed by pathology; patients without other organ tumors; and patients with informed consent signed by themselves or their family members. The mean age of included patients was 61.36±9.25 years old. Another 20 blood samples of healthy controls were collected as normal controls, with patients’ average age in the control group of 55.27±10.88 years old. Ethics has been approved by the Ethics Committee of our hospital.

### Cell culture

Glioma cells, U251, T98, LN-229, and U87, were all cultured in the culture medium containing 10% FBS and 100U/ml of penicillin/streptomycin, which were placed in a incubator with 5% CO_2_ at 37°C. 

### qRT-PCR

Patients’ plasma and cells were collected and cells were lysed by adding the TRIzol reagent. After the tissues and cells were completely dissolved, chloroform solution was mixed evenly, the upper transparent liquid was sucked into the EP tube containing isopropanolat after the low-temperature and high-speed centrifugation, in which RNA precipitation was obtained. After being washed with ethanol absolute twice, RNA was dissolved by adding double distilled water without RNA enzyme. The AMV one-step RT-PCR kit was adopted for RNA reverse transcription and PCR amplification, with 1μl of 10X one-step RT-PCR buffer, 1μg of RNA and 0.2μl of each upstream and downstream primer, 0.1μl of AMV RT, 0.1μl of RNase inhibitor, 1μl of Taq DNA polymerase, and 10μl of double steamed water without RNA enzyme for supplement. After operating, the reverse transcription was performed at 45°C for 20min, pre-denaturation at 94°C for 5min, denaturation at 94°C for 30 s, annealing at 60°C for 30 s, and extension at 72°C for 30 s, for a total of 40 cycles, as well as the final extension at 72°C for 10min. According to the internal reference gene GAPDH, the data were normalized for each parallel sample, and the relative FAM84B expression was calculated with 2^–∆∆Ct^. All the experiments were repeated three times. The primer sequences of FAM84B were F: 5′-GACCCACCTAAGTTACAAGGAAG-3′, R: 5′-GTAGAACACGGAGCATTCCAC-3′. Those of β-Actin were F: 5′-AGAAGGCTGGGGCTCATTTG-3′, R: 5′-AGGGGCCCACAGTCTTC-3′.

### Cell transfection

Glioma cells with the highest FAM84B expression were selected for FAM84B siRNA transfection. The cells in the log phase were collected with tryptase digestion and were inoculated into 6-well plates at 2×10^5^/well, which were divided into si-NC and si-FAM84B groups. After the incubation for 12h in the incubator, siRNA of each group was mixed with lip 2000 and the medium, which was added to the cells. After the culture for 6h, the medium was replaced for fresh, followed by another 48h of culture. The sequence of si-FAM84B-1 was 5′-GCAACCAGGTGGAGAAATT-3′ and of si-FAM84B-2, 5′-GGAATAGAATCATAGTTAA-3′. FAM84B expression was detected by qRT-PCR, to verify the effect of FAM84B siRNA transfection.

### Cell proliferation detection for MTT assay

The cells were collected by trypsin digestion and inoculated in 96-well plates at 1500 cells/well, with 150μl of the medium. Each group has 6 parallel samples, which were cultured in the incubator. A total of 20μl of the MTT reagent was added to each sample cell medium at 0h, 24h, 48h, and 72h of cell adhesion and mixed by gently shaking. After the incubation for 4h in the incubator, the cell culture medium was discarded, with 150μl of DMSO added, accompanied by gentle shaking for mixing. After the complete dissolution of crystals, the cell absorbance (OD) of samples at 490nm was analyzed with the full wavelength scanner.

### Flow cytometry detection for cell cycle

The cells were collected by trypsin digestion, resuspended in cold PBS, and fixed in 75% ethanol overnight. Fixed cells were washed with PBS for three times and collected by centrifugation, which were incubated with the buffer containing 50 g/ml propidium iodide (PI) and RNA enzyme at 37°C for 1h. Cell cycle distribution in each group was analyzed by flow cytometry detection.

### Western blotting

The cell plaques in each group were collected by tryptic digestion and washed in PBS for three times. Protein lysate was added to completely lyse the cells on ice, which were centrifuged at 4°C and 14000g at ultra high-speed for 30min. The centrifuged cell fragments were discarded and the protein concentration was detected with a BCA assay kit. The protein supernatant was added to the protein loading buffer and boiled in boiling water for 3min. A total of 20μg of protein sample loading was taken for gel electrophoresis to separate the target proteins. The protein was transferred to the PVDF membrane, on which the non-specific protein was sealed with the skimmed milk. After washing with TBST for three times, the PVDF membrane was incubated with target primary antibody dilution (Cyclin D1 1:1000, CDK2 1:1000, CDK4 1:1000, CDK6 1:1000, P53 1:500, P21 1:500, and GAPDH 1:1000) for 4°C, which was incubated with secondary antibody dilution (1:8000) at room temperature for 1h after washing with TBST for more three times. After washing with TBST for another three times, protein bands were exposed with the ECL kit. Images were taken with the BIORAD gel imaging system, which were processed with Image Lab3.0 software. The gray value of each protein footprint was obtained, and GAPDH was used as a reference to quantitatively analyze the gray ratio of the target protein to the internal reference in each treatment group.

## Results

### High expression of FAM84B in glioma cells

**Fig. 1:**
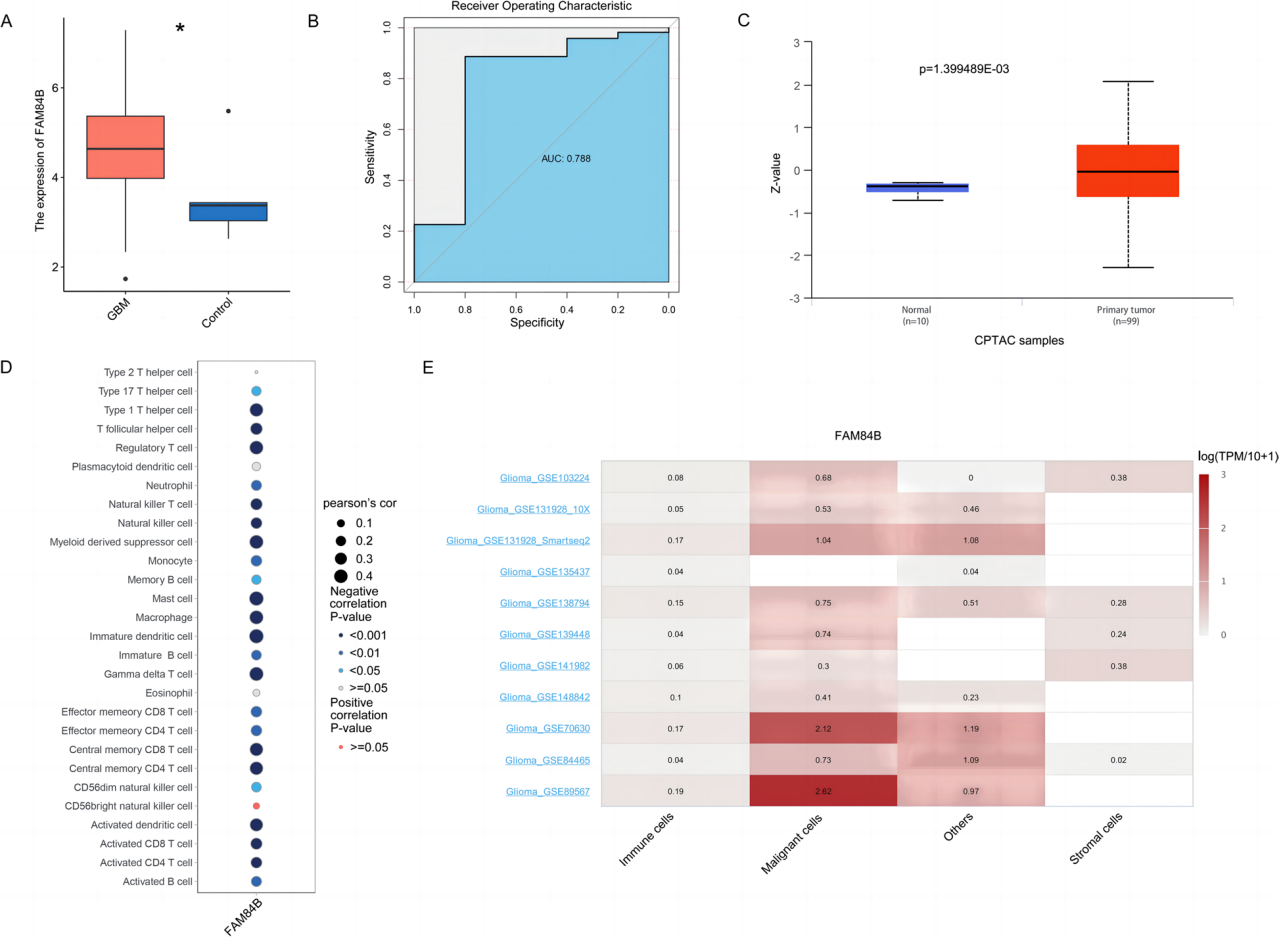
FAM84B was highly expressed in glioma patients, *P<0.05

As shown in Fig. 1A, the expression of the FAM84B gene in glioma tissue was significantly higher than that in normal brain tissue (*P* < 0.05). In addition, the ROC curve showed that FAM84B has an AUC of 0.788 (Fig. 1B). The UALCAN database analysis showed that the expressions of FAM84B protein in 90 glioma tissues were significantly higher than those in 10 normal brain tissues (*P* = 1.399489e−03) (see Fig. 1C).

The levels of immune cell infiltration in 28 gliomas were analyzed using ssGSEA. It was found that FAM84B gene expression was negatively correlated with the vast majority of immune cells (see Fig. 1D). The TISCH database analysis showed that FAM84B was highly expressed in malignant glioma cells and low in immune cells and stromal cells.

### qRT-PCR detection for FAM84B expression in glioma cells

**Fig. 2:**
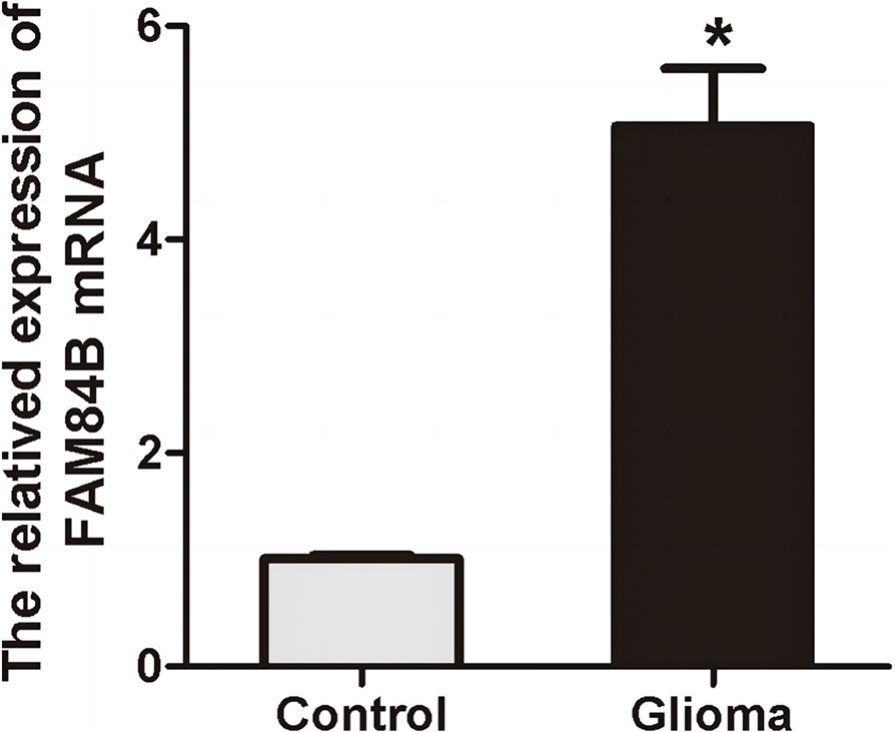
qRT-PCR detection for FAM84B expression in the serum with glioma, *P < 0.05

FAM84B mRNA expression was significantly higher in the plasma of patients with glioma than that in the serum of healthy controls as detected by qRT-PCR, *P* < 0.05, as shown in Fig. 2.

### qRT-PCR detection for FAM84B expression in glioma cells

**Fig. 3:**
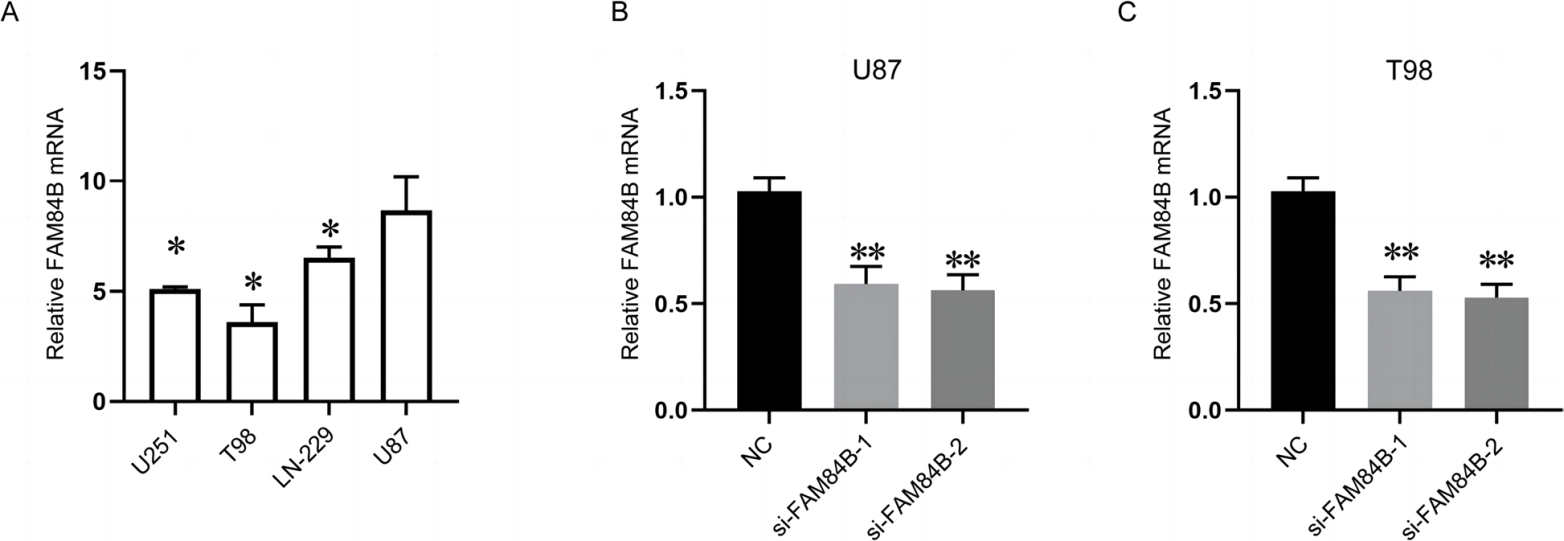
qRT-PCR detection for FAM84B expression in glioma cells. **A** FAM84B expression in the human glioma cell lines, U251, T98, LN-229, and U87; **B**, **C** FAM84B expression in U87 and T98 cells after transfection in two si-FAM84B groups and the si-NC group; *P < 0.05, **P < 0.01

FAM84B expression was detected by qRT-PCR in the human glioma cell lines, U251, T98, LN-229, and U87, showing the highest expression in U87 cells, *P* < 0.05, as shown in Fig. 3A. In order to ensure the universality of the research results, U87 and T98 cells were taken to transfect two FAM84B siRNAs for subsequent functional experiments. According to qRT-PCR, FAM84B expression after transfection of two si-FAM84B using U87 and T98 cells was significantly lower than that in the si-NC group, *P* < 0.05, as shown in Fig. 3B, C, indicating that the two FAM84B siRNAs significantly inhibited FAM84B expression in U87 and T98 cells, and can continue for subsequent functional experiments.

### Effect of interfering with FAM84B expression on the proliferation of the glioma cell lines

**Fig. 4:**
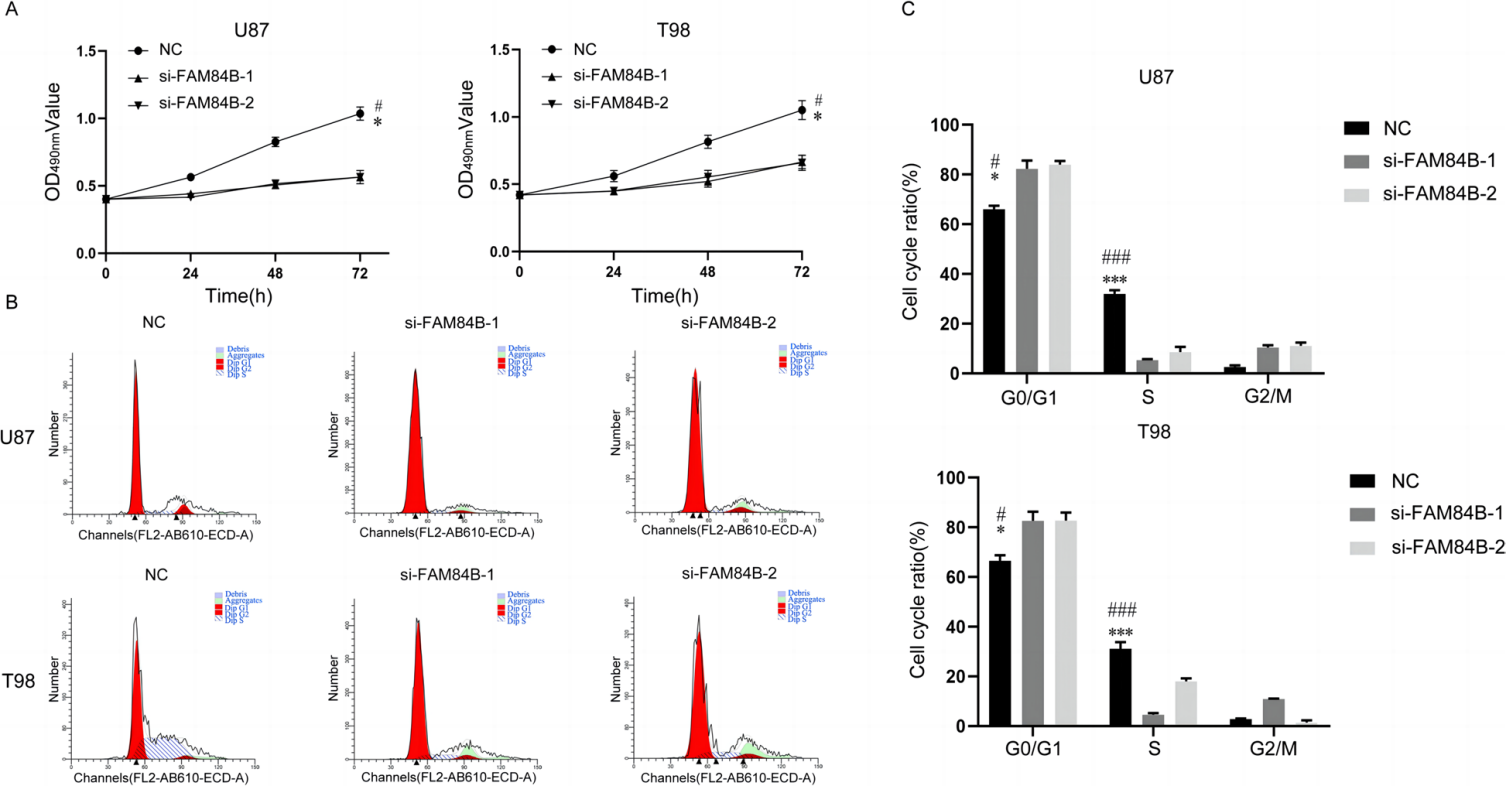
Effect of interfering with FAM84B expression on the proliferation of the glioma cell lines U87 and T98. **A** MTT detection for the effect of FAM84B on the proliferation of glioma cells; **B**, **C** flow cytometry assay for the effect of FAM84B on the cell cycle of glioma cells U87 and T98; *compared with the si-FAM84B-1 group, #compared with the si-FAM84B-2 group, *P* < 0.05

The effect of FAM84B siRNA on the proliferation of the glioma cell lines U87 and T98 as evaluated by MTT, showing a significantly decreased proliferation of U87 and T98 cells in the si-FAM84B group compared to the si-NC group, *P* < 0.05, is shown in Fig. 4A. The effect of FAM84B siRNA on the cell cycle of glioma cells U87 and T98 was detected by flow cytometry assay, showing an increased G0/G1 phase cell ratio of U87 and T98 cells in the si-FAM84B group compared to the si-NC group, *P* < 0.05, as shown in Fig. 4B and C.

### Bioinformatic analysis on FAM84B regulating the cell cycle pathways

**Fig. 5:**
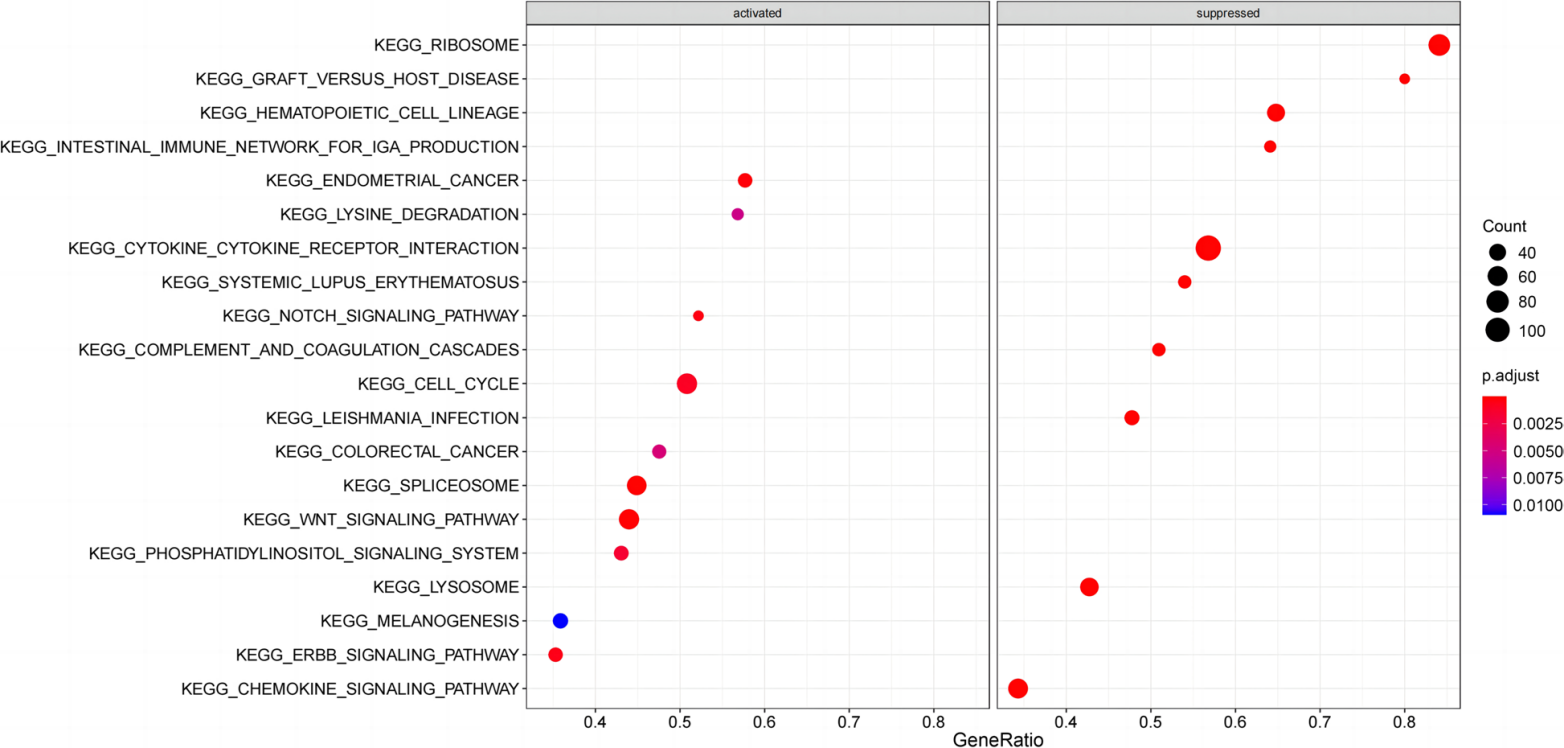
KEGG enrichment analysis showed that FAM84B can regulate the cell cycle pathways

According to FAM84B gene expression, patients with glioma were divided into upregulated and regulated FAM84B expression groups. The Bayesian analysis was performed through R software to obtain the *t* value of the whole genome, and then, FAM84B was obtained to regulate the signal pathways according to the GSEA algorithm. The analysis results showed that FAM84B can regulate the cell cycle pathways, as shown in Fig. 5.

**Fig. 6:**
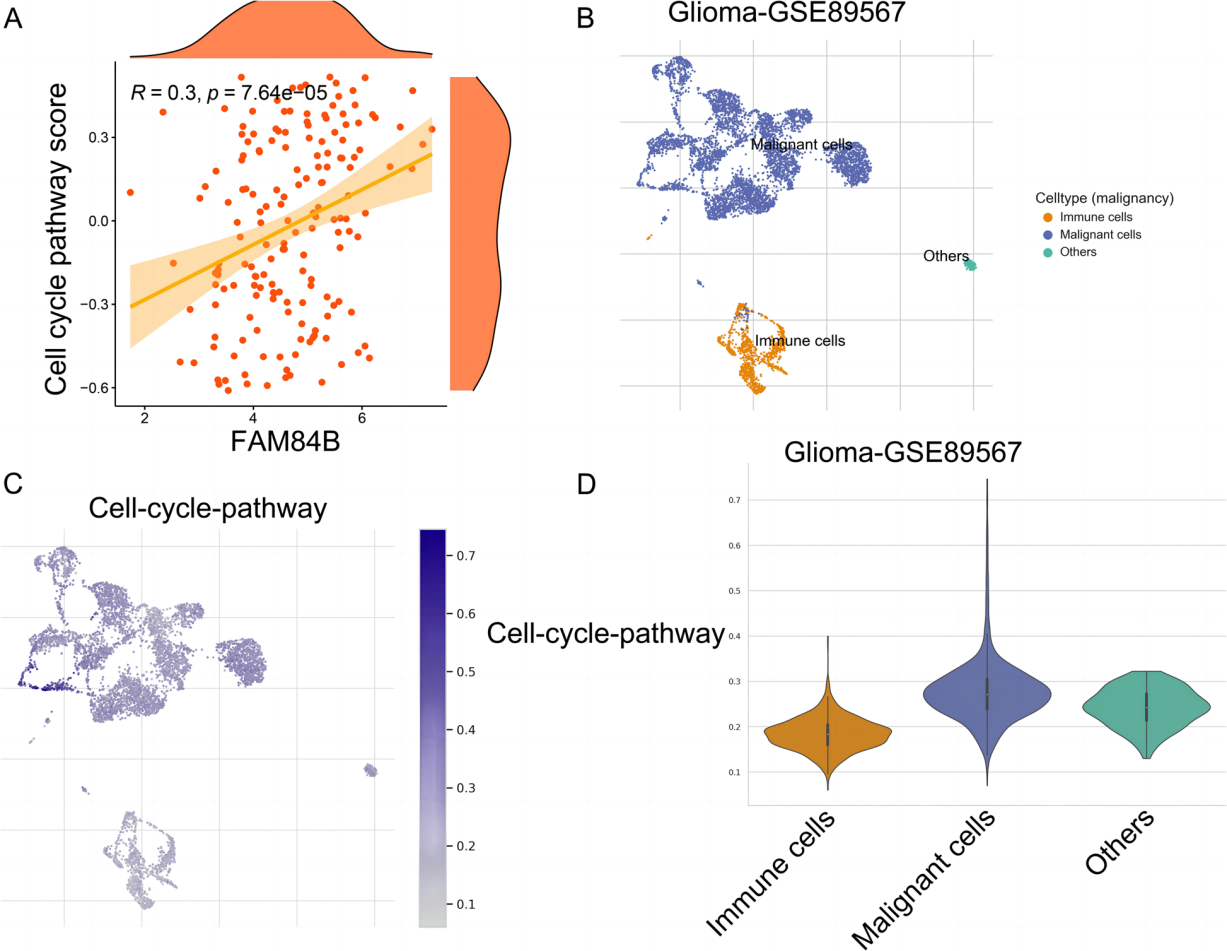
Relationship between cell cycle pathway fraction and FAM84B and its presentation in Glioma_GSE89567 data

The ssGSEA analysis was used to calculate the cell cycle pathway score in glioma. It was found that FAM84B expression was positively correlated with the cell cycle pathway score, *R*=0.3, *P*=7.64E−05 (see Fig. 6A). TISCH data showed that Glioma_GSE89567 single-cell data can be divided into malignant cells, immune cells, and other cells (see Fig. 6B). The cell cycle pathway fraction in malignant tumor cells, immune cells, and other cells are shown in Fig. 6C. We found that the cell cycle pathway fraction was the highest in malignant cells (see Fig. 6D).

### Effect of interfering with FAM84B expression on the expression of key proteins in the U87 and T98 cell cycle pathway in glioma cells

**Fig. 7:**
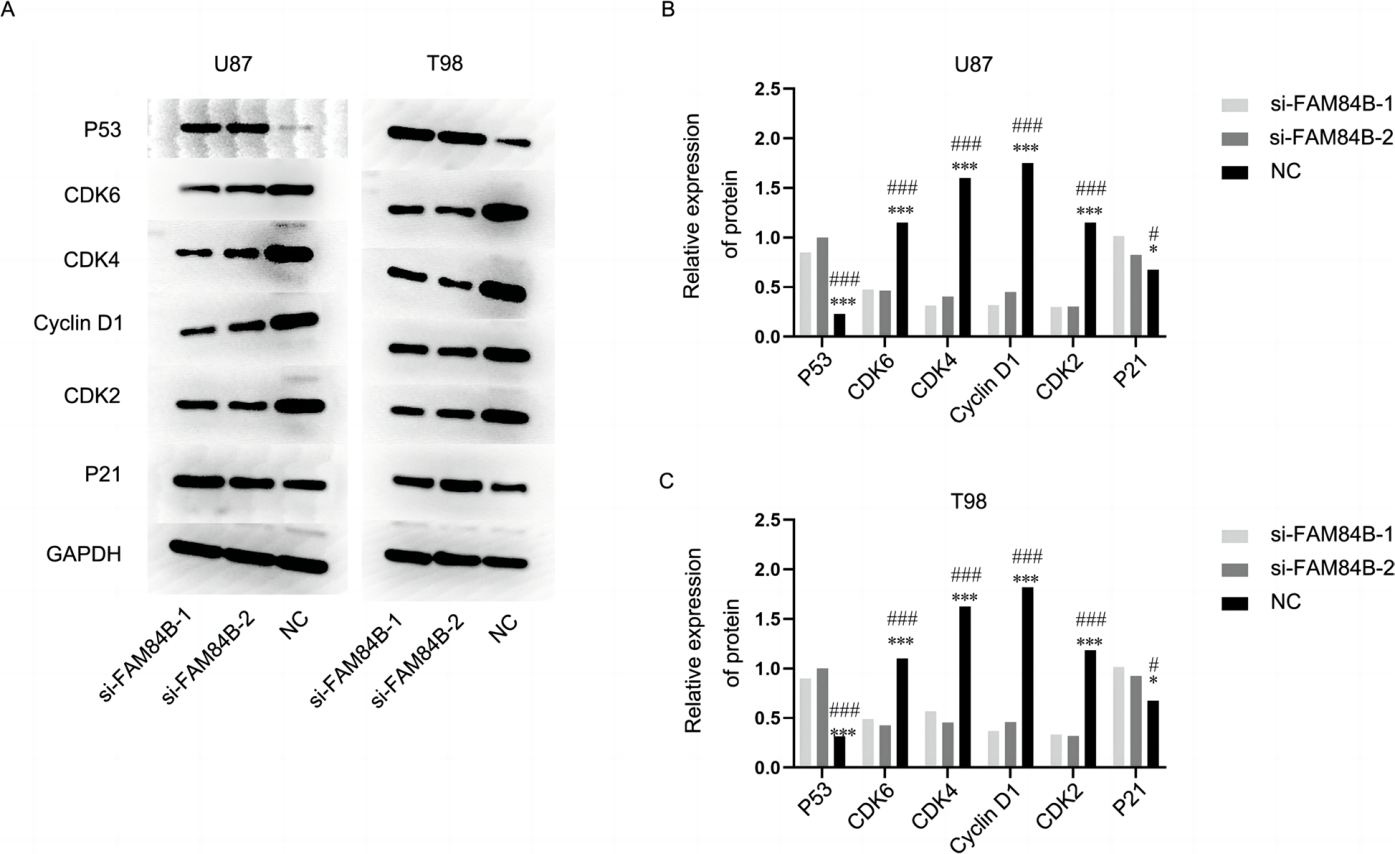
Effect of interfering with FAM84B expression on the expression of key proteins in the U87 and T98 cell cycle pathway in glioma cells. *Compared with the si-FAM84B-1 group, ^#^compared with the si-FAM84B-2 group

Western blotting results showed that the expression of key proteins in the cell cycle pathways, Cyclin D1, CDK2, CDK4, and CDK6, were decreased, and the expression of P53 and P21 proteins increased in U87 and T98 cells in both the si-FAM84B groups compared with the si-NC group, *P* < 0.05, as shown in Fig. 7.

## Discussion

As the most common intracranial tumor, glioma has the characteristics of high recurrence rate, high disability rate, and high case fatality rate. The 5-year case fatality rate is second only to pancreatic cancer and lung cancer among systemic tumors. which brings a heavy burden to society [11]. Temozolomide (TMZ) is currently the first-line chemotherapy drug for glioma treatment, which can cause alkylation of nucleophilic groups in cells, resulting in nucleobase mispairing during DNA replication and confusion in code interpretation. At the same time, bifunctional alkylating agents often combine with one guanine on each of the DNA double strands to form cross-links, hinder DNA replication, and can also damage DNA structure and function, resulting in cell division, proliferation stop, or death. But there were also a few cells that survived after DNA repair, resulting in drug resistance [12]. Tumorigenesis is a complex pathological process, and studies have shown that the change of oncogene and tumor suppressor genes is an important mechanism to promote the occurrence and development of glioma, as a new target for the treatment of glioma [13]. With the development of sequencing technologies and genome-wide association studies, multiple genetic variants increasing the risk of having got glioma have been identified, including chromosomal loci 8q24 [14]. Studies have shown that chromosomal loci 8q24 is one of the most frequently amplified areas in multiple tumor types such as colorectal cancer, breast cancer, and prostate adenocarcinoma, with an important role in the occurrence and development of tumors [15, 16].

In this paper, the GTEx and TGCA databases [17] of the GEPIA website were used to obtain and analyze the differential expression of FAM84B in normal brain tissue and glioma, which showed that FAM84B expression in glioma tissues was significantly higher than that in normal brain tissues, suggesting a possible role for FAM84B as a pro-oncogene in glioma. Zhang et al. [9] studied and found that FAM84B expression is elevated in pancreatic ductal adenocarcinoma and promotes the proliferation of tumor cells by activating the Wnt/-catenin signaling pathway, with the resistance to gemcitabine, as well as the inhibition of apoptosis. To further investigate the function of FAM84B in glioma, FAM84B expression in the glioma cell lines, U251, T98, LN-229, and U87, was first detected, and qRT-PCR showed the highest FAM84B expression in glioma cells, U87, so U87 was used as a kind of tool cells for functional experiments. U87 cells were transfected with FAM84B siRNA, and MTT assay showed that the proliferation of glioma cells, U87, significantly decreased after inhibiting FAM84B expression, which has a consistency with the studies and reports by Wang et al. [10]. They investigated the expression pattern of FAM84B in several glioma cell lines and clarified the relationship between FAM48B and the oncogenicity of glioma cells. Their results showed that FAM84B plays an important role in the proliferation, invasion, and apoptosis of glioma cells by affecting the Akt/GSK-3β/β-catenin pathway. On this basis, this study further explored the biological mechanism of FAM48B affecting cell proliferation. Through bioinformatics analysis, it was found that FAM48B is involved in the regulation of the cell cycle, which can regulate the expression level of key proteins in the cell cycle pathway in glioma cells U87, affecting the progression of the cell cycle. The cell cycle is one of the important mechanisms to regulate the cell malignant proliferation. Under normal circumstances, the cell cycle carries out an orderly periodic cycle under a series of regulatory mechanisms. When regulated by external stimulation or oncogene, the cell cycle is out of control, manifested as the cell malignant proliferation [18].

Literature review showed that Cyclin D1 promotes the transition from the G0/G1 phase to the S phase in the cell cycle by forming a complex through binding to the cyclin-dependent kinase CDK 4/6 [19, 20]. The cyclin-dependent kinase, CDK2, is a serine/threonine protein kinase that plays a key role in the G1/S transition by inducing P21 to inhibit CDK2 activity after DNA was damaged [21, 22]. In this study, interfering with FAM84B expression decreased the expression of proteins, Cyclin D1, CDK 2, CDK 4, and CDK 6, and that of P53 and P21 proteins increased in glioma cells, U87. At the same time, Tang et al. [23] showed that silencing endogenous erythropoietin (EPO) could lead to a decrease in the expression of Cyclin D1 and an increase in the expression of p27kip1 through the AKT/GSK-3β/β-catenin pathway. As a cyclin-dependent kinase (CDK) inhibitor, high-level expression of p27kip1 can arrest tumor cells in the G1 phase. Combined with our research, it is speculated that inhibiting the expression of FAM84B can affect the AKT/GSK-3β/β-catenin pathway to regulate the key proteins of the cell cycle, induce cell cycle arrest, and then inhibit the malignant proliferation of glioma cells. In this study, the target gene was regulated by transient transfection of siRNA. Although this method can be used to obtain the expression product of the gene in a short time, the exogenous gene is gradually lost as the cell divides and proliferates. The inhibitory effect gradually diminishes.

## Conclusions

In conclusion, FAM84B expression significantly increased in glioma tissues and cells, and interfering with FAM84B expression significantly inhibited the proliferation of glioma cells, and the mechanism acts through inhibiting the cell transition from G0/G1 to S phase. The research results provide a theoretical basis for FAM84B as a therapeutic target for glioma and provide new ideas for clinical drug development.

## Data Availability

The datasets used and/or analyzed during the current study are available from the corresponding author on reasonable request.
